# L’encéphalopathie de Gayet Wernicke: aspects cliniques et anomalies radiologiques

**DOI:** 10.11604/pamj.2020.36.259.14410

**Published:** 2020-08-10

**Authors:** Siham Bouchal, Naoual Bougtoub, Badr Alami, Naima Chtaou, Mustafa Maaroufi, Faouzi Belahsen

**Affiliations:** 1 Service de Neurologie, Centre Hospitalier Universitaire Hassan II, Fès, Maroc,; 2 Service de Radiologie, Centre Hospitalier Universitaire Hassan II, Fès, Maroc

**Keywords:** Encéphalopathie de Gayet Wernicke, anomalies IRM, clinique et contexte, Gayet Wernicke encephalopathy, MRI anomalies, clinical, context

## Abstract

L´encéphalopathie de Gayet Wernicke (EGW) est une urgence neurologique secondaire à une carence en thiamine (vitamine B1) le plus souvent secondaire à l´alcoolisme chronique. L´objectif de ce travail est de rappeler certaines situations cliniques évocatrices d´EGW autre que l´éthylisme et les différentes anomalies en IRM autour de 4 observations. L´âge moyen des patients était de 40 ans (2 femmes et 2 hommes). Le tableau neurologique comportait des troubles de la vigilance chez tous les patients, des troubles oculomoteurs dans 2 cas, et une ataxie cérébelleuse chez un seul patient. La notion de vomissements chroniques était notée dans 2 cas, un jeûne prolongé dans un cas et l´alcoolisme pour le dernier. L´IRM cérébrale avait révélé des anomalies évocatrices d´EGW chez tous les patients avec une prise de contraste pour un cas. Le déficit en thiamine était confirmé chez 2 patients. Dans notre contexte l´EGW semble être plus fréquente dans d´autres circonstances pathologiques autres que l´alcoolisme chronique (vomissements chroniques, dénutrition sévère, un jeûne pathologique, et chimiothérapie...). Le contexte clinique peut faire suspecter d´autres pathologies comme la thrombose veineuse cérébrale, l´accident vasculaire cérébral, ou un autre trouble métabolique mais l´IRM a permis de les écarter et de poser le diagnostic d´EGW. L´IRM cérébrale présente un intérêt primordial dans le diagnostic de l´EGW. L´absence ou le retard de la mise en route du traitement influence le pronostic.

## Abstract (en)

Gayet Wernicke encephalopathy (EGW) is a neurological emergency secondary to thiamine deficiency (vitamin B1). This is more often secondary to chronic alcoholism. The purpose of this study is to remind clinicians of some clinical signs different from ethylism, suggesting EGW as well as to report four cases characterized by different types of anomalies shown by MRI. The average age of patients was 40 years (2 females 2 males). The neurological picture was characterized by disorders of vigilance in all patients, oculomotor disorders in 2 cases, and cerebellar ataxia in one patient. Chronic vomiting was reported in two cases, prolonged fasting in the first case and alcoholism in the second case. MRI of the brain showed anomalies suggesting EGW in all patients with contrast enhancement in one case. Thiamin deficiency was confirmed in two patients. In our context EGW seems to be more frequent in pathological circumstances other than chronic alcoholism (chronic vomiting, severe malnutrition, severe starvation, and chemotherapy...). The clinical signs can suggest other pathologies such as cerebral venous thrombosis, stroke, or other metabolic disorders but MRI excluded them and allowed the diagnosis of EGW. MRI of the brain has an essential role in the diagnosis of EGW. Absence or delay in treatment may influence the prognosis.

## Introduction

L´encéphalopathie de Gayet Wernicke (EGW) est une encéphalopathie grave secondaire à une carence aiguë en thiamine (vitamine B1) qui risque d´entrainer des séquelles graves ou le décès en cas de retard diagnostic. Le présent article fait le point sur certaines situations cliniques responsables d´EGW et ses anomalies IRM articulé autour de 4 observations caractéristiques.

## Méthodes

Étude rétrospective descriptive sur 24 mois de janvier 2014 à décembre 2016 dans le Centre Hospitalier Universitaire de Fès. On a recruté 4 patients ayant un tableau clinique et radiologique compatible avec une EGW. L´IRM cérébrale des quatre patients a été réalisée sur une machine de 1,5 Tesla en coupes axiales en densité protonique, en T2 et en FLAIR en et coupes sagittales en T1. L´injection de gadolinium a été réalisée chez deux patients. Une imagerie pondérée en diffusion en échoplanar (EPI) est obtenue chez tous patients. Le dosage sanguin de la thiamine était effectué chez 2 patients.

## Résultats

**Figure 1:**
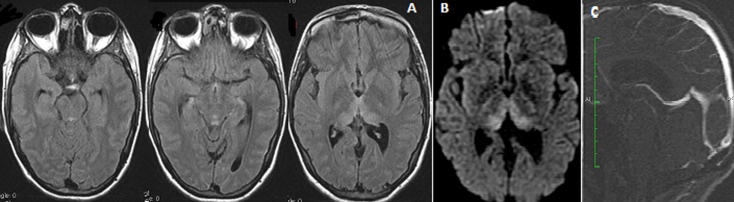
IRM cérébrale axiale FLAIR (A), diffusion (B) montrant des lésions en hypersignal bilatérales et symétriques de la région paramédiane des thalami, periacqueducale et des tubercules mamillaires. L’angio-MR veineuse (C) est normale

**Observation 1**: madame M.F âgée de 21 ans, enceinte de 18 semaines, qui présente depuis 2 mois des vomissements chroniques avec amaigrissement important, admise en réanimation pour syndrome confusionnel et tétraparésie avec réflexes ostéo-tendineux abolis. L´IRM avait révélé sur la séquence FLAIR des lésions bilatérales symétriques en hypersignal au niveau thalamique, périaqueducale, et des tubercules quadrijumeaux. L´angioMR veineuse était normale (Figure 1). Un syndrome de Gayet Wernicke compliquant des vomissements gravidiques est évoqué. Le dosage sanguin de thiamine était bas et une supplémentation en vitamine B1 était instaurée. L´évolution immédiate a été marquée par une amélioration clinique.

**Figure 2:**
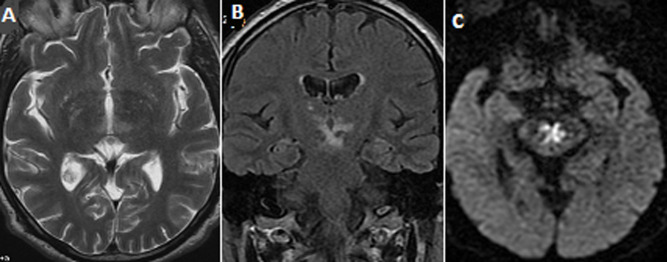
IRM cérébrale en coupe axiale T2 (A) et coronale FLAIR (B) et Diffusion (C) montrant un hypersignal mésencephalique périacqueducal et autour du 3^e^ ventricule avec restriction de la diffusion

**Observation 2:** monsieur M.E âgé de 60 ans, ayant comme antécédent le diabète type 2, le tabagisme et l´éthylisme chronique, admis pour trouble de la vigilance, avec un score de Glasgow à 12 sans autre signe neurologique de focalisation. Le scanner cérébral était normal. L´IRM avait démontré en T2, en FLAIR et en diffusion un signal hyperintense mésencephalique périaqueducale et autour de 3éme ventricule (Figure 2). Un syndrome de Gayet Wernicke secondaire à l´alcoolisme est évoqué et un traitement à base de vitamine B1 est instauré. L´évolution clinique était favorable.

**Figure 3:**
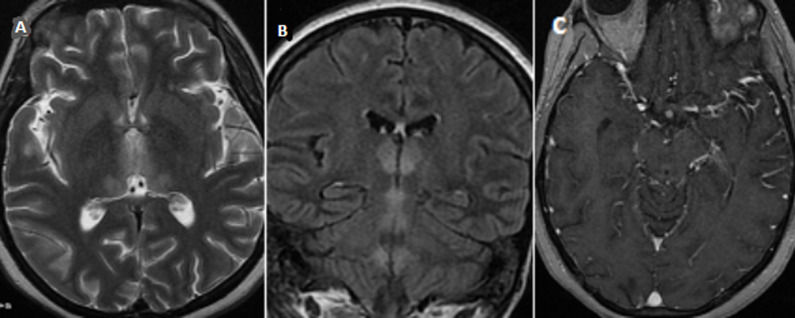
IRM cérébrale montrant un hypersignal T2 (A) et FLAIR (B) bilatéral symétriques des 2 thalamis, des régions périventriculaires, des parois du 3^e^ ventricule et des tubercules mamillaires avec discrète prise de contraste de la région périaqueducale

**Observation 3**: madame M.O âgée de 52 ans, diabétique sous insuline admise pour vomissements chroniques secondaires à une pancréatite chronique et un abcès du foie. La patiente a présenté un syndrome confusionnel avec à l´examen une ophtalmoparésie bilatérale. L´IRM a montré en séquence FLAIR et T2 un hypersignal bithalamique, périaqueducale, autour de 3^e^et 4^e^ventricules, et des tubercules mamillaires (Figure 3). Le dosage sanguin de thiamine était bas.

**Figure 4:**
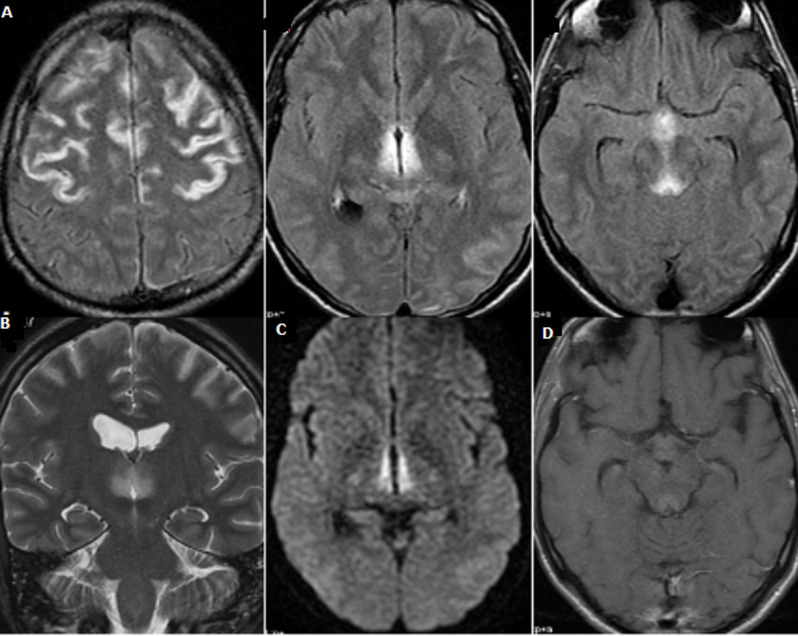
IRM cérébrale en coupe axiale FLAIR (A) et coronale T2 (B) montrant un hypersignal cortical frontal bilatéral symétrique, périaqueducal, des thalami. Il y a une restriction de la diffusion (C) des lésions thalami et discrète rehaussement périaqueducal (D)

**Observation 4**: monsieur MM âgé de 30 ans, qui présente suite à une grève de faim prolongée un syndrome confusionnel et une ophtalmoparésie bilatérale. L´IRM a révélé un hypersignal en T2, en FLAIR cortical frontal bilatéral, autour du 3^e^ventricule, et des tubercules mamillaires (Figure 4) évoquant une EGW.

## Discussion

La thiamine ou vitamine B1 est un cofacteur de plusieurs réactions enzymatiques notamment dans la voie secondaire de la glycolyse et dans le cycle de Krebs qui est une voie métabolique cruciale de la production de l´énergie chimique sous la forme de l´adénosine triphosphate (ATP) indispensable au fonctionnement cellulaire [1,2]. Elle joue aussi un rôle de neurotransmetteur, en potentialisant les effets de l´acétylcholine. Au niveau tissulaire, la thiamine est captée par les cellules et transformée en une forme coenzymatique active [1-3]. Les besoins journaliers de thiamine chez un adulte sont de 1-2 mg de thiamine. Les réserves de cette vitamine du corps ne sont que de 30 à 50 mg, de sorte que toute condition de malnutrition d´une durée supérieure à 3 ou 4 semaines peut entraîner un épuisement complet de ce stock et entrainer un dysfonctionnement cellulaire grave [2]. Le métabolisme des régions périventriculaires est particulièrement sous dépendance de cette vitamine, ce qui explique la prédominance de la souffrance cellulaire à ce niveau en cas de déficit en B1. L´encéphalopathie de Gayet Wernicke (EGW) traduit la carence aiguë et massive en vitamine B1 par diminution de son apport, de son absorption ou par sa mauvaise utilisation [1,3]. L´encéphalopathie de Gayet Wernicke (EGW) est décrite par Wernicke en 1881. Elle touche le plus souvent l´homme que la femme, classiquement entre 30 et 70 ans [2]. Elle est révélée cliniquement par une triade, qui associe des troubles neuropsychiques (syndrome confusionnel, apathie, bradypsychie, hypersomnie), des troubles oculomoteurs et des troubles de l´équilibre, en rapport avec un syndrome vestibulaire central et un syndrome cérébelleux [1]. La triade n´est cependant complète que dans 8 à 30% des cas ce qui rend le diagnostic d´EGW difficile, retardé et parfois même en post mortem [1,3]. Lorsque les troubles oculomoteurs sont présents ils sont très évocateurs, mais ils ne se voient que chez 15 à 29% des cas [3-5]. Le nystagmus (mouvements oculaires involontaires) ou l´ophtalmoplégie (paralyse du regard conjugué) sont des signes classiques, alors que l´atteinte intrinsèque est relativement rare [3,6]. Parfois une polyneuropathie peut être associée à l´atteinte centrale.

La cause la plus fréquente de la carence en vitamine B1 est l´alcoolisme. Cette carence est secondaire aux complications de la cirrhose hépatique avec la malabsorption intestinale et la malnutrition qui en résulte. Autres que l´alcool, la carence vitaminique B1 peut également survenir dans d´autres situations associant une malnutrition ou une diminution de l´absorption telles que la chirurgie gastro-intestinale, les vomissements gravidiques, la chimiothérapie, le SIDA, l´anorexie mentale, le jeûne prolongé, la dénutrition, la nutrition parentérale, et perfusion intraveineuse prolongée du sérum glucosé [2]. L´imagerie cérébrale par résonance magnétique (IRM) peut révéler des anomalies classiques de l´EGW [1,3] et elle est largement sensible par rapport au scanner avec une spécificité qui dépasse les 90% [2]. Les anomalies retrouvées sont des hypersignaux dans les séquences pondéré T2, FLAIR et parfois diffusion. Elles sont symétriques, et siègent au niveau des noyaux thalamiques postéro-médiaux, de part et d´autre du 3éme ventricule, des corps mamillaires et de la région périaqueducale [7-9]. Il existe aussi des localisations atypiques telle que la tête du noyau caudé, les noyaux lenticulaires, et le cortex mais qui sont toujours associées aux anomalies classiques. Un rehaussement dans ces mêmes zones est rapporté aussi dans la littérature [7]. La diffusion peut révéler un hypersignal qui peut être soit en rapport avec un œdème vasogénique ou cytotoxique. La séquence ADC permet de différencier entre les deux mécanismes [2]. L´intérêt supplémentaire de l´IRM cérébrale est d´éliminer les autres diagnostics puisque le tableau clinique n´est pas spécifique. Un retard de la mise en route d´un traitement par la thiamine en cas d´EGW peut mettre en jeu le pronostic vital et fonctionnel. Pourtant, les protocoles thérapeutiques curatifs sont hétérogènes et non consensuels. Une revue de la littérature en 2016 concernant les articles publiés durant une période de 15 ans sur le traitement préventif et curatif d´EGW à partir de 2000, a permis d´établir des algorithmes de prescription de thiamine en cas de suspicion d´EGW. Cette étude propose également des protocoles de prévention dans les situations à risque de produire un déficit en thiamine. La posologie de thiamine recommandée est de 500 mg IV 3 fois/jour pour une durée de 3 à 5 jours, suivie, en cas d´amélioration au traitement initial, de 250 mg en intraveineux par jour pour un minimum de 3 à 5 jours supplémentaires [10].

## Conclusion

L´EGW une urgence diagnostique et thérapeutique et la supplémentation en vitamine B1 par voie parentérale doit être instauré en urgence devant un tableau clinique et radiologique évocateur sans attendre la confirmation biologique afin d´éviter des séquelles neurologiques parfois graves.

### Etat des connaissances sur le sujet

- L´EGW est une urgence diagnostique et thérapeutique;
- L´antécédent d´alcoolisme est le plus évocateur de l´EGW et le plus incriminé comme cause.

### Contribution de notre étude à la connaissance

- Les vomissements prolongés et le jeûne prolongé peuvent être la cause d´EGW: y penser et traiter tôt, même en l´absence de notion d´alcoolisme;
- En plus de l´atteinte radiologique classique de l´EGW, la prise de gadolinium et l´atteinte du cortex sont aussi possibles.
